# Supplementary figures and images for: Keep Garfagnina alive. An integrated study on patterns of homozygosity, genomic inbreeding, admixture and breed traceability of the Italian Garfagnina goat breed
Source: PLoS One. 2021 Jan 15;16(1):e0232436. doi: 10.1371/journal.pone.0232436 (PMC7810337; doi:10.1371/journal.pone.0232436)

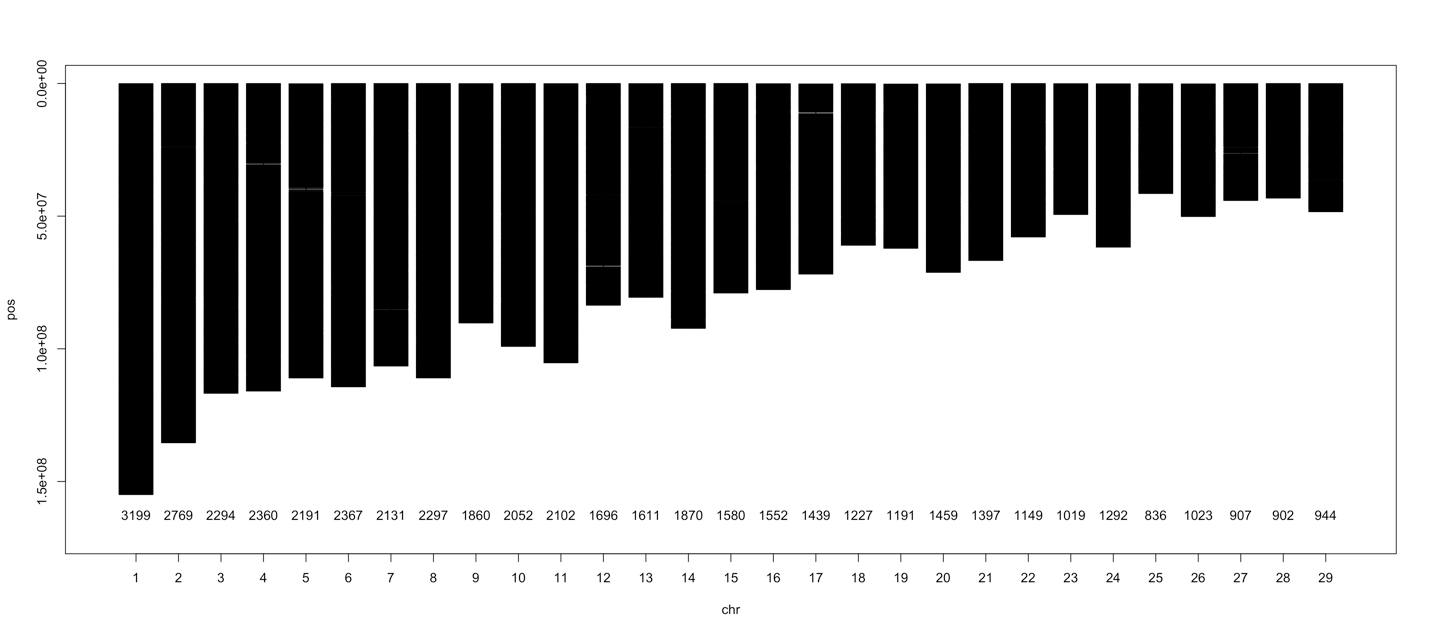


**S1 Fig**

Supplement: S1 Fig — (DOCX) [file pone.0232436.s001.docx]

a)
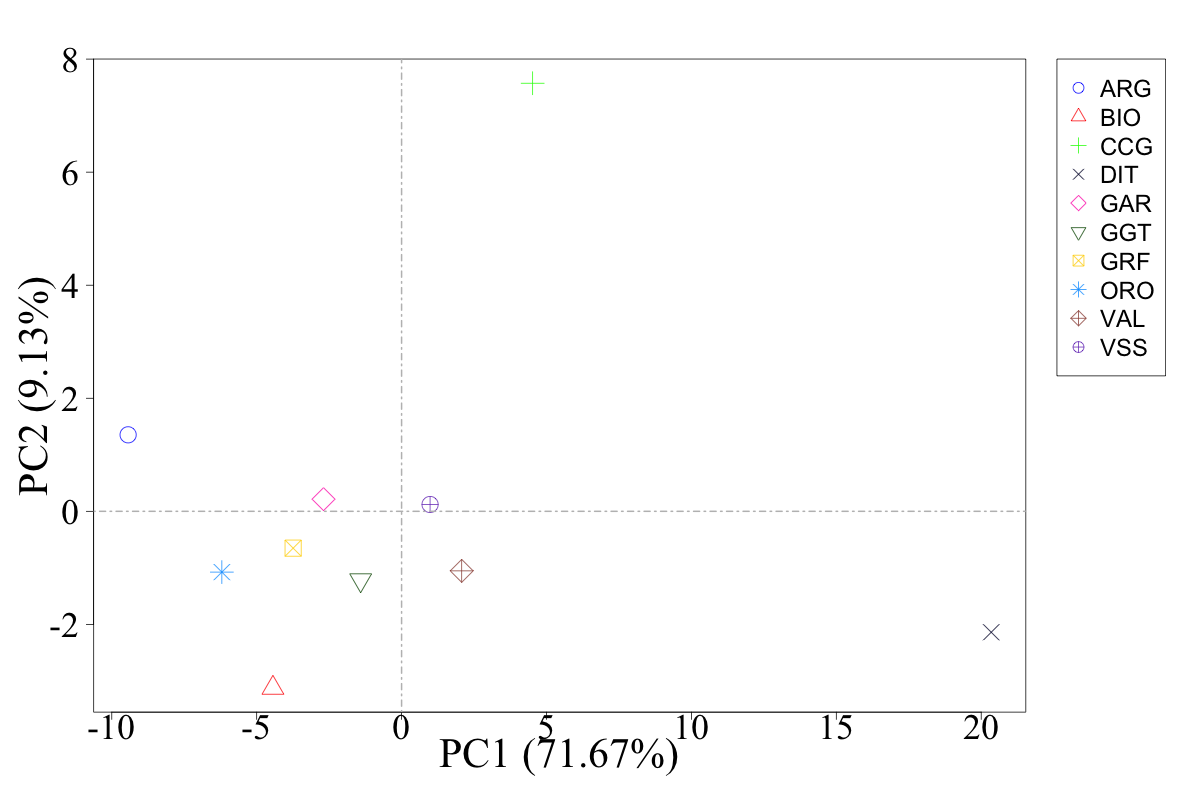


b)
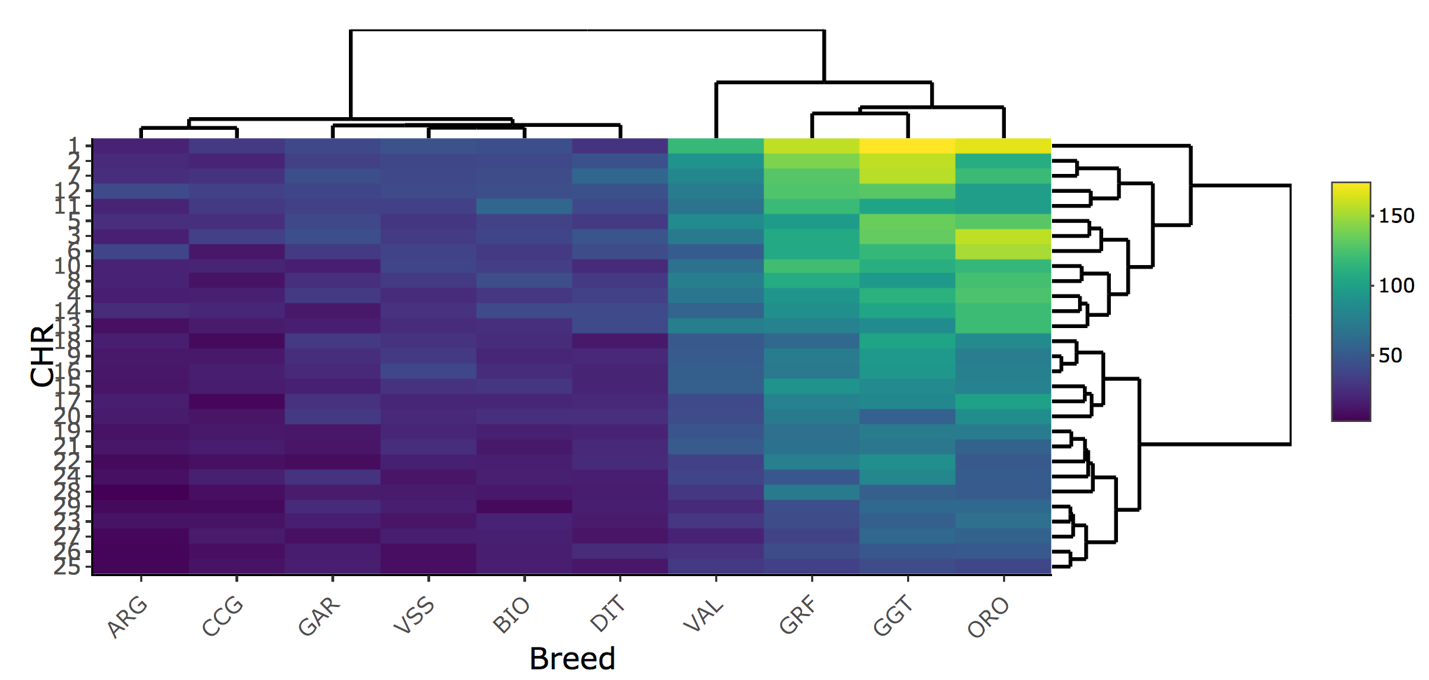


**S2 Fig**

Supplement: S2 Fig — ARG: Argentata dell’Etna; BIO: Bionda dell’Adamello; CCG: Ciociara Grigia; DIT: Di Teramo; GAR: Garganica; GGT: Girgentana; GRF: Garfagnina; ORO: Orobica; VAL: Valdostana and VSS: Valpassiria. (DOCX) [file pone.0232436.s002.docx]

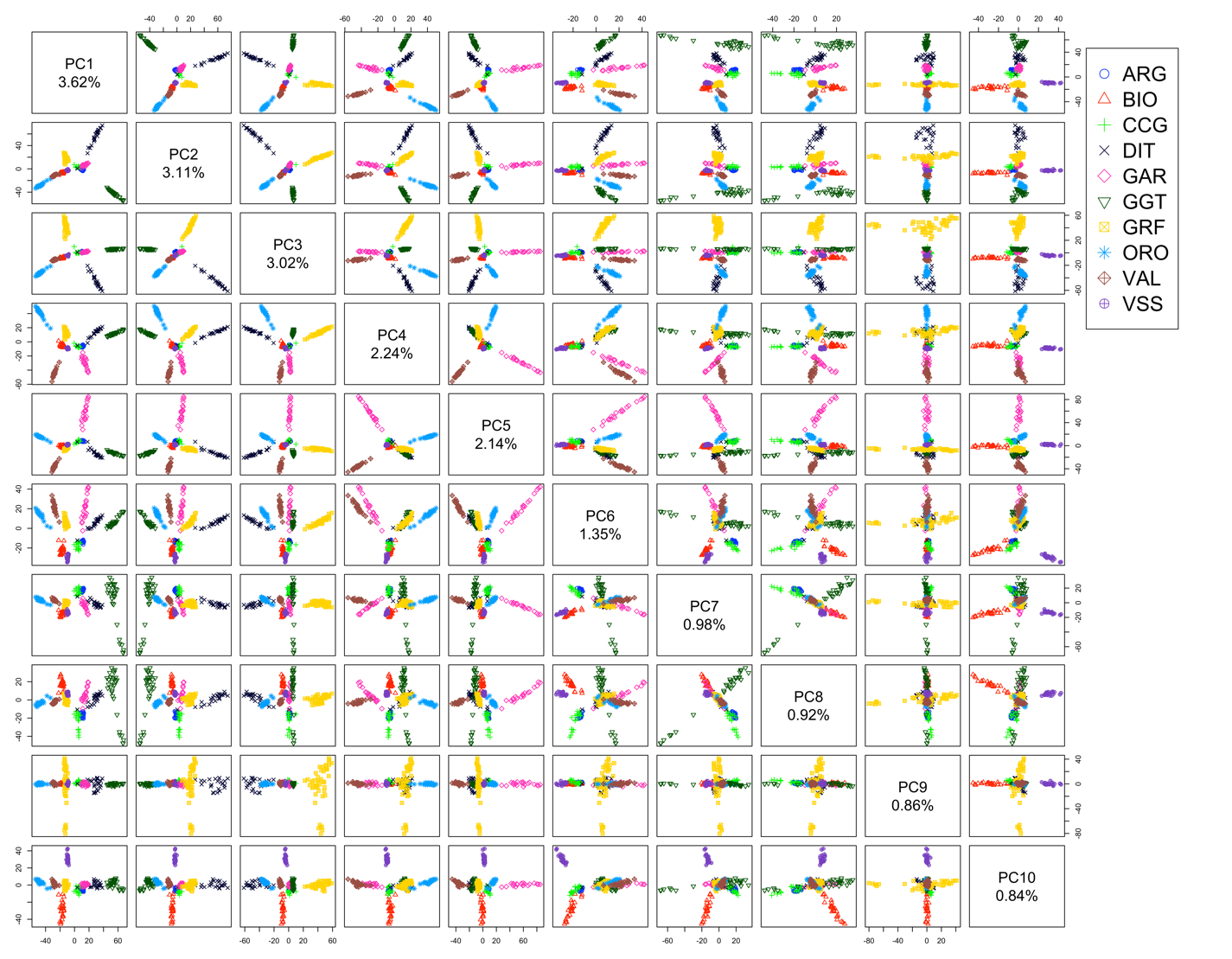


**S3 Fig**

Supplement: S3 Fig — 1Singular value decomposition was applied on the matrix of genotypes. (DOCX) [file pone.0232436.s003.docx]

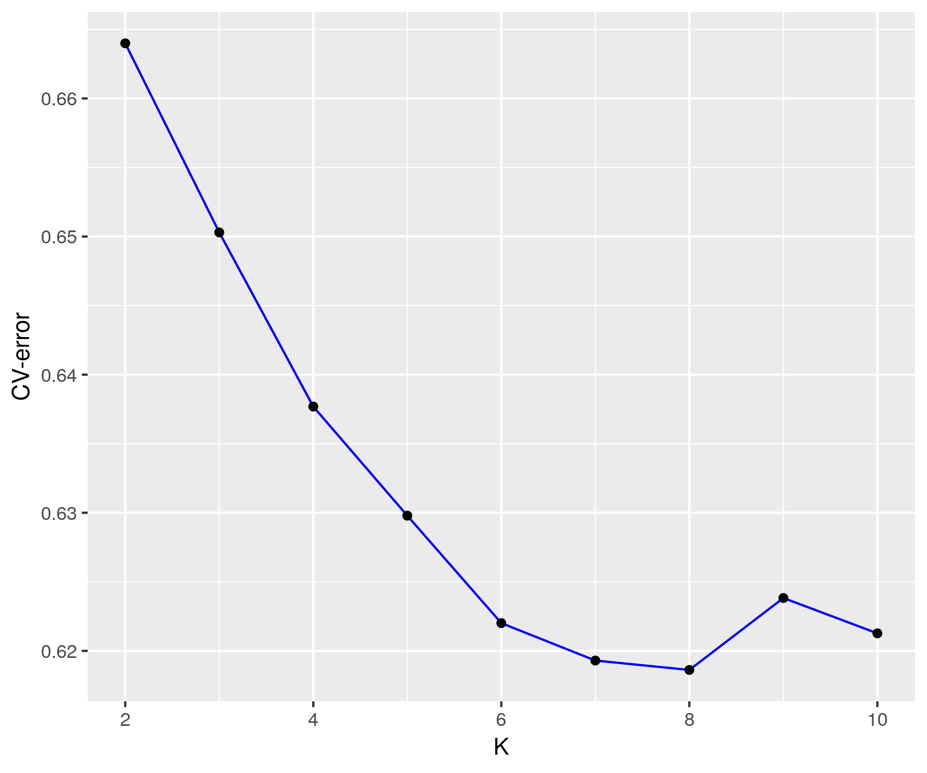


**S4 Fig**

Supplement: S4 Fig — (DOCX) [file pone.0232436.s004.docx]

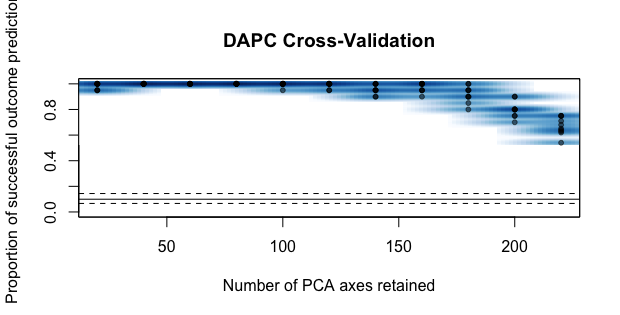


**S5 Fig**

Supplement: S5 Fig — (DOCX) [file pone.0232436.s005.docx]
