# Supplementary material for: Keep Garfagnina alive. An integrated study on patterns of homozygosity, genomic inbreeding, admixture and breed traceability of the Italian Garfagnina goat breed
Source: PLoS One. 2021 Jan 15;16(1):e0232436. doi: 10.1371/journal.pone.0232436 (PMC7810337; doi:10.1371/journal.pone.0232436)
Supplement: S1 Table — ARG: Argentata dell’Etna; BIO: Bionda dell’Adamello; CCG: Ciociara Grigia; DIT: Di Teramo; GAR: Garganica; GGT: Girgentana; GRF: Garfagnina; ORO: Orobica; VAL: Valdostana and VSS: Valpassiria. (DOCX) [file pone.0232436.s006.docx]

**S1 Table**

| **Class of length of ROH** | **ARG** | **BIO** | **CCG** | **DIT** | **GAR** | **GGT** | **GRF** | **ORO** | **VAL** | **VSS** |
| --- | --- | --- | --- | --- | --- | --- | --- | --- | --- | --- |
| 0-2 | 78.06% | 63.76% | 50.60% | 35.30% | 50.00% | 47.32% | 53.80% | 55.33% | 55.05% | 57.29% |
| 2-4 | 12.04% | 15.72% | 15.12% | 13.53% | 25.89% | 27.48% | 24.82% | 27.78% | 19.71% | 17.84% |
| 4-8 | 7.10% | 12.29% | 16.94% | 19.31% | 15.62% | 15.13% | 13.43% | 11.85% | 11.90% | 12.50% |
| 8-16 | 1.51% | 6.02% | 10.69% | 18.33% | 5.62% | 7.49% | 6.00% | 4.16% | 7.94% | 7.94% |
| > 16 | 1.29% | 2.21% | 6.65% | 13.53% | 2.88% | 2.57% | 1.96% | 0.89% | 5.39% | 4.43% |
