# Supplementary material for: Keep Garfagnina alive. An integrated study on patterns of homozygosity, genomic inbreeding, admixture and breed traceability of the Italian Garfagnina goat breed
Source: PLoS One. 2021 Jan 15;16(1):e0232436. doi: 10.1371/journal.pone.0232436 (PMC7810337; doi:10.1371/journal.pone.0232436)
Supplement: S2 Table — ARG: Argentata dell’Etna; BIO: Bionda dell’Adamello; CCG: Ciociara Grigia; DIT: Di Teramo; GAR: Garganica; GGT: Girgentana; GRF: Garfagnina; ORO: Orobica; VAL: Valdostana and VSS: Valpassiria. (DOCX) [file pone.0232436.s007.docx]

**S2 Table**

| **Breed** | **Min** | **Max** | **Mean** | **Median** | **SD** | **CV** |
| --- | --- | --- | --- | --- | --- | --- |
| ARG | 0.007 | 0.100 | 0.018 | 0.013 | 0.018 | 1.01 |
| BIO | 0.004 | 0.169 | 0.045 | 0.028 | 0.041 | 0.92 |
| CCG | 0.002 | 0.146 | 0.053 | 0.040 | 0.050 | 0.93 |
| DIT | 0.004 | 0.292 | 0.103 | 0.110 | 0.081 | 0.78 |
| GAR | 0.001 | 0.325 | 0.056 | 0.021 | 0.080 | 1.45 |
| GGT | 0.058 | 0.346 | 0.143 | 0.125 | 0.073 | 0.51 |
| GRF | 0.003 | 0.177 | 0.069 | 0.061 | 0.035 | 0.51 |
| ORO | 0.027 | 0.281 | 0.137 | 0.134 | 0.047 | 0.35 |
| VAL | 0.034 | 0.360 | 0.122 | 0.084 | 0.099 | 0.81 |
| VSS | 0.012 | 0.182 | 0.054 | 0.033 | 0.049 | 0.91 |
