# Supplementary material for: Keep Garfagnina alive. An integrated study on patterns of homozygosity, genomic inbreeding, admixture and breed traceability of the Italian Garfagnina goat breed
Source: PLoS One. 2021 Jan 15;16(1):e0232436. doi: 10.1371/journal.pone.0232436 (PMC7810337; doi:10.1371/journal.pone.0232436)
Supplement: S4 Table — CVSS: semi-supervised CV, where some GRF goats are present in model training; CVUS: unsupervised CV, where GRF breed had no representative goats in model training. (DOCX) [file pone.0232436.s009.docx]

**S4 Table**

| **CV repetition** | **CV_SS_** | **CV_US_** |
| --- | --- | --- |
| 1 | 50 | 40 |
| 2 | 20 | 60 |
| 3 | 30 | 40 |
| 4 | 50 | 20 |
| 5 | 40 | 40 |
| 6 | 70 | 40 |
| 7 | 70 | 60 |
| 8 | 50 | 60 |
| 9 | 20 | 40 |
| 10 | 10 | 40 |
