# Supplementary material for: Keep Garfagnina alive. An integrated study on patterns of homozygosity, genomic inbreeding, admixture and breed traceability of the Italian Garfagnina goat breed
Source: PLoS One. 2021 Jan 15;16(1):e0232436. doi: 10.1371/journal.pone.0232436 (PMC7810337; doi:10.1371/journal.pone.0232436)
Supplement: S5 Table — ARG: Argentata dell’Etna; BIO: Bionda dell’Adamello; CCG: Ciociara Grigia; DIT: Di Teramo; GAR: Garganica; GGT: Girgentana; GRF: Garfagnina; ORO: Orobica; VAL: Valdostana and VSS: Valpassiria. (DOCX) [file pone.0232436.s010.docx]

**S5 Table**

| **Breed** | **CV_US__1** | **CV_US__2** | **CV_US__3** | **CV_US__4** | **CV_US__5** | **CV_US__6** | **CV_US__7** | **CV_US__8** | **CV_US__9** | **CV_US__10** |
| --- | --- | --- | --- | --- | --- | --- | --- | --- | --- | --- |
| ARG | 0 | 0 | 0 | 0 | 0 | 0 | 0 | 0 | 0 | 0 |
| BIO | 0 | 0 | 0 | 0 | 0 | 0 | 0 | 0 | 0 | 0 |
| CCG | 34 | 38 | 34 | 38 | 32 | 32 | 38 | 38 | 33 | 32 |
| DIT | 4 | 0 | 4 | 0 | 6 | 6 | 0 | 0 | 5 | 6 |
| GAR | 0 | 0 | 0 | 0 | 0 | 0 | 0 | 0 | 0 | 0 |
| GGT | 0 | 0 | 0 | 0 | 0 | 0 | 0 | 0 | 0 | 0 |
| GRF | 0 | 0 | 0 | 0 | 0 | 0 | 0 | 0 | 0 | 0 |
| ORO | 0 | 0 | 0 | 0 | 0 | 0 | 0 | 0 | 0 | 0 |
| VAL | 0 | 0 | 0 | 0 | 0 | 0 | 0 | 0 | 0 | 0 |
| VSS | 0 | 0 | 0 | 0 | 0 | 0 | 0 | 0 | 0 | 0 |
