# Supplementary material for: Keep Garfagnina alive. An integrated study on patterns of homozygosity, genomic inbreeding, admixture and breed traceability of the Italian Garfagnina goat breed
Source: PLoS One. 2021 Jan 15;16(1):e0232436. doi: 10.1371/journal.pone.0232436 (PMC7810337; doi:10.1371/journal.pone.0232436)
Supplement: Attachment — Submitted filename: Response_To_Reviewers.docx [file pone.0232436.s011.docx]

Title: **Keep Garfagnina alive. An integrated study on patterns of homozygosity, genomic**

**inbreeding, admixture and breed traceability of the Italian Garfagnina goat breed.**

AU: We thank the reviewers for their constructive review. Please find below our responses to the points raised. All our responses are preceded by “AU” (and in blue). Track changes were used in the manuscript. Extra changes, not requested by the reviewers, are highlighted in cyan. We hope to find the new version of the manuscript suitable for publication in PLOS ONE.

Looking forward to hearing from you.

Sincerely,

Dr. Christos Dadousis

**Academic Editor**

Dear Dr. DADOUSIS,

Thank you for submitting your manuscript to PLOS ONE. After careful consideration, we feel that it has merit but does not fully meet PLOS ONE’s publication criteria as it currently stands. Therefore, we invite you to submit a revised version of the manuscript that addresses the points raised during the review process.

There is a number very valid points the reviewers mention that should be implemented in the revised MS to improve readability and clarity. Please pay attention to the discussion to avoid repetitions or results that are mentioned earlier. These points focus on the presentation of the study rather than the data or analysis and should be easy to implement.

AU: We have tried to address all points raised by the reviewers. The Discussion part has been improved for clarity, following the reviewers’ suggestions. The manuscript has been further checked by two external colleagues, acknowledged in the Acknowledgments (L497-502), to improve readability and correct syntax errors.

**Reviewer 1**

The paper describes SNP data analyses of an Italian goat breed (Garfagnina). More particularly, the authors have carried out ROH, admixture, PCA and DAPC analyses. In my opinion, data analysis has been sufficiently rigorous, and I especially like the part about DAPC. DAPC is a quite complicated method, and the authors went to lengths to avoid drawing incorrect conclusions from inappropiate settings.

In contrast, in my opinion the presentation of the results is currently not sufficient to justify publication. Points which should be addressed are:

- The text contains many syntax errors. I listed a few of these errors below, but this list represents a random subselection only. One example is consistent misuse of the word 'the'. I suggest all authors to proofread the text prior to resubmission.

AU: We have been throughout the manuscript and corrected syntax errors. Moreover, the manuscript has been checked from two external colleagues who are acknowledged in the Acknowledgments (L497-502).

- The figures are not ready to publish and should be polished prior to publication. For example, the admixture plot is enormous, the pca/dapc plots are very hard to read (soft colours), and the legends should be inflated to make them readable.

AU: Indeed, the Figures in the printed pdf version are not in good quality. However, the Figures we have uploaded in PLOS ONE are of high quality. We assume this is a result during the creation of the final pdf file. We are at the availability of the editor to solve the problem.

- Number of figures should be reduced, or alternatively figures should be compiled into multi tile figures. For example: the first 4 figures are all on ROHs. Why not summarize the results in one figure?

AU: Both recommendations have been followed. The Figures of ROH have been placed together and the total number of Figures has been reduced either by excluding or moving Figures to supplements.

- Last but not least: for me the overall question, and how the performed analyses address this question, is not clear and should be clarified.

AU: Several parts have been added or re-arranged for clarity, mainly in the Discussion section (e.g., L81-87, L316-340). Moreover, we added a new subsection proposing measures to be taken for the conservation of the breed, supported by appropriate scientific literature (L438-480).

Minor comments

Title:

What is ‘genomic inbreeding’?

AU: The term genomic inbreeding refers to estimates of inbreeding coefficients of individuals by genomic data rather than pedigree (L84-85). The term is widely known in animal and plant breeding, hence we would like to keep it. As an example, we provide with a list of papers making use of the term “genomic inbreeding” (Gomez-Raya et al., 2015; Mastrangelo et al., 2016; Schiavo et al., 2020; VanRaden et al., 2011). However, following the reviewer’s suggestions other changes in terminology have been done throughout the manuscript for clarity, such as the “genomic background” in the following comment.

Gomez-Raya, L., Rodríguez, C., Barragán, C., Silió, L., 2015. Genomic inbreeding coefficients based on the distribution of the length of runs of homozygosity in a closed line of Iberian pigs. Genet. Sel. Evol. 47, 81. https://doi.org/10.1186/s12711-015-0153-1

Mastrangelo, S., Tolone, M., Gerlando, R.D., Fontanesi, L., Sardina, M.T., Portolano, B., 2016. Genomic inbreeding estimation in small populations: evaluation of runs of homozygosity in three local dairy cattle breeds. animal 10, 746–754. https://doi.org/10.1017/S1751731115002943

Schiavo, G., Bovo, S., Bertolini, F., Tinarelli, S., Dall’Olio, S., Nanni Costa, L., Gallo, M., Fontanesi, L., 2020. Comparative evaluation of genomic inbreeding parameters in seven commercial and autochthonous pig breeds. Anim. Int. J. Anim. Biosci. 14, 910–920. https://doi.org/10.1017/S175173111900332X

VanRaden, P.M., Olson, K.M., Wiggans, G.R., Cole, J.B., Tooker, M.E., 2011. Genomic inbreeding and relationships among Holsteins, Jerseys, and Brown Swiss. J. Dairy Sci. 94, 5673–5682. https://doi.org/10.3168/jds.2011-4500

Abstract:

What do you mean with: ‘genomic background’?

AU: This term is also familiar to the animal/plant breeding community. However, we agree with the reviewer that the term is too general, although the same stands for other terms, such as the very definition of a “breed” (see L329-333 in the manuscript). Hence, we tried to avoid the usage of this term throughout the manuscript, which was mainly replaced by the term “genetic diversity”.

Line 21-22:

I don’t understand the opening sentence: ‘local breeds are recognized as a rule of rural land protection’?

AU: This part has been rephrased (L24-25). Indeed, E.U. and F.A.O. have increased interest in the conservation of local breeds. Apart from the risk of extinction, due to small numbers in many local populations, local breeds have been recognized as a measure of rural land protection and sustainability. The reasons are that local breeds do not only relate to human culture but also they fit better to the local environment. For example, the greater grazing potential of local small ruminants compared to their cosmopolitan competitors, acts as a measure of protection against avalanches in winter and fire outbreaks during the summer period. In addition, it is very common that local breeds are linked to niche and PDO products with geographic origin. These are described in the first paragraph of the Introduction (L30-42).

Line 32:

Why not just: ‘Grazing is a cost-effective, nonpolluting…’

AU: Changed as suggested (L34).

Line 34:

Alternative to what?

AU: The sentence has been rephrased (L36).

Line 37:

Incorrect sentence formulation: ‘provide with an alternative’

AU: The sentence has been rephrased (L36).

Line 41-43:

Incorrect sentence

AU: The sentence has been rephrased (L44-46).

Line 45:

Incorrect: ‘at critical situation’

AU: Changed to “in a critical situation” (L48-49).

Line 79:

Specific you are talking about goats used in this study

AU: Ethics statement relates to the samples of Garfagnina we have sampled.

Line 95-97:

inconsistent sentence construction

AU: AU: The sentence has been rephrased (L106-108).

Line 105:

Incorrect to say analysis were conducted in a R package

AU: The sentence has been rephrased (L118).

Line 115:

Surrounding genes only? Not overlapping genes?

AU: In this part we mean that a search in the whole region (1Mb up/downstream) was followed. Because the word “surrounding” might be misleading to the reader, it has been removed. The sentence has been rephrased to “…were detected and genes located within ± 1 Mbp were annotated …” (L130-131).

Line 127:

What do you mean with ‘traceability’?

AU: Changed to “breed traceability” throughout the manuscript (e.g., L9, 144, 409, 434).

Line 204:

Even though you specified the details in the methods sections, it would still be helpful for the reader to see the definition of ROH mentioned in this table or in the caption. Also I wonder whether the absolute number of ROHs is really informative. Would it not be more informative to present percentages of the genome, or percentage of number SNPs which are part of the ROHs?

AU: We have introduced a brief explanation of ROH in the Introduction (L83-86). A clear definition of ROH consists from several parts reported in the M&M (L120-122). Hence, we believe that none of these could fit in a Table. Regarding the results on ROH, we have included together with the absolute number, the average number, the length per chromosome, and the distribution per ROH class (Fig 1), and the percentage per length class and breed (S1 Table). In addition, the genomic inbreeding summarizes by definition the length of ROH over the total autosomal length per goat, summed over all goats per breed.

Line 260:

What do you mean with: ‘complement with’?

AU: Admixture and PCA are two approaches very similar and complementary to each other. PCA is widely used to identify structure in the data and to distinguish groups between the samples. In that sense, the objective of PCA is to summarize (dis)similarities over the different groups in the data rather than the individual itself. On the other hand, admixture is focusing on the individuals; it provides with the probabilities of each individual to be clustered in one of the pre-defined group ancestries.

Line 321:

Consistent incorrect use of ‘the’. For example: it is incorrect to say: ‘following from the above’

AU: We would like to thank the reviewer for this observation. We have been throughout the manuscript to correct for syntax errors. Moreover, the manuscript has been checked from two external colleagues who are acknowledged in the Acknowledgments (L497-502).

**Reviewer 2**

This study investigated the genomic background of Garfagnina goats, a local goat breed reared in the hills and mountains of the Northwestern Tuscan Apennine area in Central Italy. Despite its cultural and agroeconomic importance, the Garfagnina breed currently faces the risk of extinction and would certainly benefit from a breeding scheme assisted by genomic information. Thus, the research developed here has its value and the methods used are scientifically sound. However, the manuscript doesn’t live up to the standard expected from a full research article and needs to be improved before it is accepted for publication. I listed below some problems that I think should be addressed.

The major problem I see in the manuscript is that a great portion of the Discussion is not serving to its purpose. The Discussion section should be used to put the results into some context, to draw conclusions, and to show their significance and implications. However, the first two paragraphs of discussion are only repeating information that were already stated in the Introduction and Materials & Methods, just using different words.

AU: Following your suggestions, we have focused in re-writing the Discussion. A new part has been added at the beginning of the Discussion (L316-340). Moreover, we added a new subsection proposing measures to be taken for the conservation of the breed, supported by appropriate scientific literature (L438-480). Overall, the new adds contribute in clarity and scientific significance of this work.

Another example of that in the Discussion is its section on runs of homozygosity. That whole portion (lines 330-379) can be reduced to a single table of genes present in frequent RoH regions and moved to the Results section. The Discussion should contain what the finding of these genes suggests in the context of the Garfagnina breed.

AU: The first paragraph is discussion on previous studies (L351-359). Moreover, the part between L367-384 describes the gene functionality and reports findings from previous studies. Report on the SNP name and position was included to connect the ROH results with gene annotation. Hence, we do believe that this part has been correctly assigned in the Discussion. We would like to keep this part as is in the manuscript.

Moreover, in the Discussion section on population stratification and ancestry, the authors reiterate methodological aspects of the study already described in the Materials & Methods. This redundancy is irrelevant here. Only in the last paragraph, the authors start to build on what should have been the focus of this section since its beginning.

For the reasons mentioned above, I believe that the Discussion needs a major overhaul.

AU: As mentioned above, a series of changes has been done in the Discussion, improving readability, clarity and the overall quality of the manuscript. We would like to thank the reviewer and hope that we have positively addressed the comment.

Minor comments

- The text is generally well written, but some grammar mistakes are noticeable throughout the text.

- I don’t see a point in showing PCA plots of PC1 x PC2 (Fig. 7a) and PC1 x PC6 (Fig. 7b) while skipping the other principal components (PC3–PC5), specially when the percentage of explained variance is so small. In my opinion, if you don’t have a specific reason for doing that, it is enough to show only the plot for PC1 x PC2 (or a three-dimensional plot with PC1 x PC2 x PC3) in the main text. You can show plots for additional principal components in the supplementary material, if you wish.

AU: The number of Figures have been reduced based on comments of Reviewer 1. Only the PC1-PC2 plot has been kept in the manuscript. However, we disagree that only the first 2-3 PCs are important. Each eigenvector captures specific variation of the original data. This is the reason why, for example, in many human GWAS studies (multiple linear regression without a relationship matrix) a large number of PCs is kept in the model to correct for population structure. Moreover, we have extended our research into the first 10 PCs. As it is shown, the 9^th^ PC identifies a subgroup of 6 goats in GRF. Not surprisingly, this is in agreement with the admixture analysis (L405-408).

- The same can be said about the admixture plots. I don’t see any benefits in showing only K=4 (Fig. 8a) and K=8 (Fig. 8b) in the manuscript. It makes more sense to show the results for all K as a supplementary figure and show only K=8 in the main text, since it is the value of K supported by the cross-validation.

AU: Only admixture results at K=8 have been kept.

- I also suggest you use a more contrasting color palette for the figures. Particularly in Fig. 8, it is hard to distinguish the ancestry components for some breeds.

AU: A second plot, averaging the results by breed, has been added (Fig 5b). Indeed, the Figures in the printed pdf version are not in good quality. However, the Figures we have uploaded in PLOS ONE are of high quality. We assume this is a result during the creation of the final pdf file. We are at the availability of the editor to solve the problem.
