# Supplementary material for: Keep Garfagnina alive. An integrated study on patterns of homozygosity, genomic inbreeding, admixture and breed traceability of the Italian Garfagnina goat breed
Source: PLoS One. 2021 Jan 15;16(1):e0232436. doi: 10.1371/journal.pone.0232436 (PMC7810337; doi:10.1371/journal.pone.0232436)
Supplement: Attachment — Submitted filename: Response_To_Reviewers.docx [file pone.0232436.s012.docx]

Title: **Keep Garfagnina alive. An integrated study on patterns of homozygosity, genomic**

**inbreeding, admixture and breed traceability of the Italian Garfagnina goat breed.**

AU: We would like to thank again the reviewers and the editor for their constructive review. Please find below our responses to the points raised. All our responses are preceded by “AU” (and in blue). Track changes were used in the manuscript. Extra changes, not requested by the reviewers, are highlighted in cyan. We hope to find the new version of the manuscript suitable for publication in PLOS ONE.

Looking forward to hearing from you.

Sincerely,

Dr. Christos Dadousis

**Academic Editor**

Dear Dr. DADOUSIS,

Thank you for submitting your manuscript to PLOS ONE. After careful consideration, we feel that it has merit but does not fully meet PLOS ONE’s publication criteria as it currently stands. Therefore, we invite you to submit a revised version of the manuscript that addresses the points raised during the review process.

This revised manuscript is a much improved version, and I thank the authors for their changes.

Reviewer 1 has new suggestions and comments. The authors should read these over and consider their potential to improve the manuscript, addreessing them as they see fit. Some are simple wording changes/clarifications that can be easily addressed and should be, while others are a matter of personal preference (line 192, line 399) and finally some require some more effort, for example the comment on figure 5. I do not think the authors need to make all changes in order for the paper to be acceptable for publication, and I leave it up to them to decide which ones they want to follow up on.

AU: We would like to thank the editor for the positive evaluation given to our work.

However, there are two main points that need to be addressed before acceptance:

1. Data access: PLOSONE and all PLOS journals require authors to make all data necessary to replicate their study’s findings publicly available without restriction at the time of publication. When specific legal or ethical restrictions prohibit public sharing of a data set, authors must indicate how others may obtain access to the data. Although the data is available in ENA, the identifier is not contained anywhere in the actual manuscript text to the best of my ability to find it. Please fix this.

AU: The genotypes of the Garfagnina goats have been deposited on dryad (doi:10.5061/dryad.jwstqjq73). Data availability has been added in the text on L653-655.

2. Both reviewers and I are in agreement that the figures are not quite ready for publication yet. First, multipanel figures should be consolidated into single files by the authors. In keeping with reviewer 2's comments, I recommend, but do not demand, that they replot all figures using the sample colour palettes; I notice they've tried to match the ggplot default to the R rainbow palette, but using a single colour scheme through the paper will be easier for readers! I also recommend, but again do not demand, that they use different colour schemes for 1c, such that any given colour means the same throughout the manuscript.

AU: We have replotted all figures with different colour schemes while keeping the same colour per breed throughout the manuscript. The multipanel Figures are always uploaded as single files into the system.

For the PCA plot in figure 4 I encourage (but once again, do not demand) the authors to increase the size of some of the plotting symbols, and consider their choice of colours, to make sure they're accessible to a broad audience. Likewise, I appreciate the effort that has gone into Supp Fig 3 but given the size of the plot, some of the green groups are hard to distinguish from each other - again, worth considering a slightly more distinctive colour scheme. The rcolorbrewer palettes `set1` or `dark2` (plus black and another colour) might be worth considering.

AU: We have replotted Fig 4 increasing the size of the plotting symbols. As above mentioned, we have also changed the colours to better distinguish the different breeds.

3. Finally, while reading the submission I noticed some typographical/wording points:

line 49: (all of which from Europe and Caucasus) should be (all of which are from Europe and Caucasus) or (all from Europe and Caucasus). Additionally, it should be 'the Caucasus', both here and in line 48.

AU: Changed as suggested (L50-51).

line 153: "a maximum number of 300 PCs were tested" The authors have a total of 260 samples, so how are there 300 PCs to test? I believe this is a PCA done on a samples x genotype matrix, so the max number of PCs should be 260? Please clarify this.

AU: Thank you very much for this observation, that was a typo and has been corrected (L. 157).

line 211: "For GRF, an excess of frequent ROH (more than 45% in the GRF samples analyzed)" Just clarifying that this means the ROH was seen in over 45% of GRF samples, both here and in other places where the same term is used? In that case, I recommend rewording to "high-frequency" or "common".

AU: Changed as suggested (L 12, 15, 134, 215, 366).

line 336: "This, difficulty, poses" should be "This difficulty poses"

AU: Changed as suggested (L 341)

line 402: "the distinguished and" I think the authors might mean "distinct" instead of distinguished?

AU: Changed as suggested (L 407).

line 442: "GRF i) owes a distinct" I think the authors here mean "has" or "possesses" instead of owes?

AU: Changed as suggested (L 445)

**Reviewer 1**

The authors have addressed all my comments raised in the first revision round, and therefore I have no reason not to accept the paper in the current state. I still think though that the discussion is a bit shallow and that the results could be presented in more concise plots.

AU: We would like to thank the reviewer for the general positive evaluation given to our work.

Rereading the paper, I had some additional remaining questions and further suggestions for improvement prior to final submission:

Line 4. ‘and analyzed together’

I suggest clarifying here that the data for the 214 goats of the 9 other breeds was not newly generated but taken instead from an online database. This was not initially clear to me.

AU: Changed as suggested (L 5-6).

Line 10: For GRF, an excess of ROH 11 (more than 45% in GRF samples) was detected on CHR 12 at, roughly 50.25-50.94Mbp 12 (ARS1 assembly), which spans the CENPJ (centromere protein) and IL17D (interleukin 13 17D) genes.

Why is it informative to share these specific ROH-details in the abstract?

AU: This is also part of the result. Moreover, it is very common to look for genes in ROH analysis that might be related to selection (natural or artificial). The genome assembly is reported because it is strictly linked to the genomic region identification (it is common that between different genome assemblies there might be discrepancies in terms of is re-arrangement of variants/genes within a chromosome or even across chromosomes). Hence, we would like to keep this part in the abstract.

Line 17: Overall, our results support the identification of GRF as a distinct native Italian goat breed.

Maybe the authors could specify to which analyses ‘overall’ refers to. I suppose particularly admixture analysis and DAPC analyses. The identified ROH in GRF was also present in DIT, and hence does not seem to support identification of GRF as a distinct breed. Perhaps it could be specified in the abstract that DAPC and admixture analyses were conducted to assess the relationship of GRF to other breeds, and that ROH analyses were conducted to assess the genetic diversity of the breed (?).

AU: Changed as suggested (L 11-12, 83-84). We would like to keep the term “Overall” on L19.

Line 41:

Check four double spaces throughout document (you could use the replace function in Word to replace all double spaces with single spaces).

AU: Thank you very much for this observation. We made the changes throughout the manuscript.

Line 87: Overall, our results suggest a distinct genetic pool of GRF

And what do the results suggest about the genetic diversity?

AU: The sentence has been extended (L 91) to include also genetic diversity of GRF.

Line 121

Why these parameters? Did you assess the sensitivity of the outcome to the parameter settings? At least a justification is required. In general I am not a big fan of presenting ROH analyses for one parameter setting only, but I am aware many studies do so.

AU: We would like to thank the reviewer for this comment. Indeed, we are aware from literature and own experience of the sensitivity of ROH analyses. In this work we used these parameters that could be considered as “well-established” for this SNP-panel and in line with previous works reporting Froh with the 9 complementary breeds used in our study (L 362-364). We believe that the integrated analysis of admixture, PCA, ROH and DAPC was adequate to provide with a concrete outcome to address our basic question: could we consider GRF as a distinct goat genetic pool in Italy?

ROH analyses provided information of genomic inbreeding and common homozygous segments (with different lengths). Moreover, a comparative analysis was followed, taking into account all breeds. Given that i) genomic inbreeding can be measured with various formulas (e.g, four measures reported in plink, ROH based on sliding windows or consecutive runs and at least one method of constructing GRM) with a range of correlations among these methods and ii) the sensitivity of ROH is not only related to parameters used for defining a ROH but also on SNP QC (MAF, LD and HWE) this type of analysis would be extremely extended and out of the scope of this work.

Moreover, please note that the same question goes also for DAPC for which we decided to perform an extensive analysis.

Line 192. Summary results of the detected ROH regions as either total counts or averaged 193 based on the number of samples per breed are presented in Table 2 and Fig 1a, 194 respectively.

This sentence can be deleted. Describe the main results in the text and at the end of the sentences cite the relevant figures.

AU: We would like to keep this sentence since it directly provides to the reader important information to follow ROH results.

Line 197.

Why is the distribution per chromosome relevant for the story line?

AU: The distribution of chromosome is relevant because: i) different chromosomes have different SNP densities (S1 Fig) and ii) because a frequent number of ROH on a specific chromosome might be linked with natural or artificial selection.

Line 202-203.

What does the correlation between chromosome length and total ROH length tell about the causal mechanisms behind the ROHs? Simply a chance effect of random distribution of segregating sites across the genome? If so, does it really inform about inbreeding, or is it in fact a measure of genetic diversity?

AU: Please, see our reply above. Regarding the relationship between inbreeding and genetic diversity, these factors are inter-related and difficult to distinguish, since inbreeding is already a measure of genetic diversity. In general, ROH caused by inbreeding tend to be distributed unevenly over the genome, with a different distribution of numbers and sizes of ROH for each chromosome (Pemberton et al, 2012).

Pemberton, T.J.; Absher, D.; Feldman, M.W.; Myers, R.M.; Rosenberg, N.A.; Li, J.Z. Genomic patterns of homozygosity in worldwide human populations. Am. J. Hum. Genet. 2012, 91, 275–292.

Line 317-322

Relevant information?

AU: We find as relevant information to report the general distribution of goats and would like to keep this part in the manuscript. Moreover, this part is used to open the Discussion section.

Line 367-397

What is the supposed relation between the ROH and the presence of genomic features? Background selection? I reckon it is not unlikely to detect genomic features in a 2 Mb region. In other words: the presence of genomic features within a ROH is perhaps irrelevant.

AU: ROH that are located in specific genomic regions and shared among several individuals are thought to be potential signs of selection (please see literature below).

Metzger, J.; Karwath, M.; Tonda, R.; Beltran, S.; Águeda, L.; Gut, M.; Gut, I.G.; Distl, O. Runs of homozygosity reveal signatures of positive selection for reproduction traits in breed and non-breed horses. BMC Genom. 2015, 16, 764.

Ablondi, M.; Viklund, Å.; Lindgren, G.; Eriksson, S.; Mikko, S. Signatures of selection in the genome of Swedish warmblood horses selected for sport performance. BMC Genom. 2019, 20,717.

Nolte, W.; Thaller, G.; Kuehn, C. Selection signatures in four German warmblood horse breeds: Tracing breeding history in the modern sport horse. PLoS ONE 2019, 14, 1–25.

Pemberton, T.J.; Absher, D.; Feldman, M.W.; Myers, R.M.; Rosenberg, N.A.; Li, J.Z. Genomic patterns of homozygosity in worldwide human populations. Am. J. Hum. Genet. 2012, 91, 275–292.

Line 399-436

Results section rather than discussion section

AU: We made slight changes in this part, but we would like to keep this subheading, since a comparison of the results obtained from different methods is discussed, information that we find interesting. Regarding DAPC, a discussion on the robustness of the model is provided. This information is not reported in the Results.

Figure 5.

Why not show the outcome for multiple K-values?

AU: That was a request by Rev2 in review round 1:

“The same can be said about the admixture plots. I don’t see any benefits in showing only K=4 (Fig. 8a) and K=8 (Fig. 8b) in the manuscript. It makes more sense to show the results for all K as a supplementary figure and show only K=8 in the main text, since it is the value of K supported by the cross-validation.”

**Reviewer 2**

The manuscript has improved and I'm overall content with the reformulation of the discussion, which was my major concern. However, regarding the quality of the figures, which was also pointed by reviewer 1, the issue is not the resolution of the image but the colors used. We have access to the original image files uploaded by the authors and even in the high resolution images it is hard to distinguish between different breeds in some cases. For instance, I can not easily differentiate two of the three shades of green used in Figure 5 and I'm not color blind. Apart from that, I think the study deserves to be published but the authors must work with the Editor to polish the images before publication..

AU: We would like to thank the reviewer for the positive evaluation given to our work. We have replotted all figures with different colour schemes while keeping the same colour per breed throughout the manuscript.
